# The prevalence of suicidal ideation identified by the Edinburgh Postnatal Depression Scale in postpartum women in primary care: findings from the RESPOND trial

**DOI:** 10.1186/1471-2393-11-57

**Published:** 2011-08-03

**Authors:** Louise M Howard, Clare Flach, Anita Mehay, Debbie Sharp, Andre Tylee

**Affiliations:** 1 King's College London, Health Service and Population Research Department, Institute of Psychiatry, London, UK; 2 Academic Unit of Primary Health Care, School of Social and Community Medicine, University of Bristol, Bristol, UK

## Abstract

**1 Abstract:**

**1.1 Background**

Suicide is a leading cause of perinatal maternal deaths in industrialised countries but there has been little research to investigate prevalence or correlates of postpartum suicidality. The Edinburgh Postnatal Depression Scale is widely used in primary and maternity services to screen for perinatal depressive disorders, and includes a question on suicidal ideation (question 10). We aimed to investigate the prevalence, persistence and correlates of suicidal thoughts in postpartum women in the context of a randomised controlled trial of treatments for postnatal depression.

**1.2 Methods**

Women in primary care were sent postal questionnaires at 6 weeks postpartum to screen for postnatal depression before recruitment into an RCT. The Edinburgh Postnatal Depression Scale (EPDS) was used to screen for postnatal depression and in those with high levels of symptoms, a home visit with a standardised psychiatric interview was carried out using the Clinical Interview Schedule-Revised version (CIS-R). Other socio-demographic and clinical variables were measured, including functioning (SF12) and quality of the marital relationship (GRIMS). Women who entered the trial were followed up for 18 weeks.

**1.3 Results**

9% of 4,150 women who completed the EPDS question relating to suicidal ideation reported some suicidal ideation (including hardly ever); 4% reported that the thought of harming themselves had occurred to them sometimes or quite often. In women who entered the randomised trial and completed the EPDS question relating to suicidal ideation (n = 253), suicidal ideation was associated with younger age, higher parity and higher levels of depressive symptoms in the multivariate analysis. Endorsement of 'yes, quite often' to question 10 on the EPDS was associated with affirming at least two CIS-R items on suicidality. We found no association between suicidal ideation and SF-12 physical or mental health or the EPDS total score at 18 weeks.

**1.4 Conclusions**

Healthcare professionals using the EPDS should be aware of the significant suicidality that is likely to be present in women endorsing 'yes, quite often' to question 10 of the EPDS. However, suicidal ideation does not appear to predict poor outcomes in women being treated for postnatal depression.

**Trial registration**

Current Controlled Trials ISRCTN16479417.

## 1 Background

Suicide is a leading cause of maternal deaths in the perinatal period in industrialised countries [1,2]. Suicide is more common in people with suicidal thoughts [3] and suicidal thoughts are therefore a cause for concern when elicited by health professionals. Outside of the perinatal period, being female and having major depression are associated with the highest risk of acting on suicidal ideas [4] but there is some evidence that compared with non-pregnant populations, women in the antenatal and postnatal period are at lower risk, with risk being highest in women with severe disorders [5]. This may be due to concern for the unborn child being a protective factor or may reflect that women with a high risk of suicide are less likely to get pregnant. The relationship between suicidal thoughts and suicidal acts in the postpartum period is not clear but it is prudent to assume that suicidal thoughts are a marker of increased risk of suicide [5].

There has been little research to investigate correlates of suicidality in the postpartum period, but in primary care suicidal ideation is associated with functional impairment, psychiatric comorbidity, increased health service use and subjective distress [6-8]. A review of studies of suicidality in the perinatal period found the prevalence in the postnatal period to vary from 4% (in Finland) to 15% (in India) [5]; all of these studies used the Edinburgh Postnatal Depression Scale (EPDS) [9] which includes a specific item on self harming thoughts on a four point scale 'never', 'hardly ever', 'sometimes', 'yes quite often'. However in all but one study, the study population was relatively small (< 900 women) and no other measure of suicidality was used to compare with the EPDS measure. A more recent study used the Beck Depression Inventory (BDI9) [10] in a population based sample of married women and found endorsement of the self harm question in 8.3% of 386 women [11]; in their multivariate analysis of its correlates, only a positive BDI was retained as a risk factor for suicidality but they acknowledged that the study may not have had the statistical power to detect correlates due to the limited sample size. There is therefore limited information on risk factors associated with an EPDS measure of suicidality or persistence of such suicidality. Previous studies have also not examined whether suicidal ideation is associated with worse outcome.

The Edinburgh Postnatal Depression Scale (EPDS) [9] is a widely used tool in primary care and community maternity services to screen for depressive disorders in the perinatal period [12]. There is growing evidence that it also identifies anxiety disorders [13]. Although there is some controversy about whether and when to use the EPDS in screening for postnatal mental disorders [14,15] it is still used internationally in the primary care setting. We used the EPDS to identify women in the community who were possibly depressed, for recruitment into a randomised controlled trial comparing antidepressants and non - directive counselling in the treatment of postnatal depression (the RESPOND trial) [16]. This enabled us to investigate the prevalence of suicidal thoughts in postpartum women, the persistence and correlates of suicidal thoughts in postpartum women participating in a treatment trial, and to compare the EPDS measure of suicidality with the more comprehensive assessment used as part of the Clinical Interview Schedule-Revised version (CIS-R) [17].

We aimed to:

1) determine the prevalence of suicidal ideation as measured by the EPDS in a primary care population of women at 6-8 weeks postpartum screened for postnatal depressive symptoms before being offered the opportunity to enter the RESPOND trial, and determine the persistence of suicidal ideation at 18 weeks postpartum in those that did enter the trial;

2) validate the EPDS measure of suicidal ideation using the more detailed Clinical Interview Schedule- Revised version (CIS-R) measure of suicidality;

3) determine risk factors associated with the EPDS measure of suicidality in women participating in RESPOND;

4) examine whether suicidal ideation is associated with worse outcome (quality of life; depressive symptoms on EPDS; quality of marital relationship) at follow-up during the RESPOND trial.

We hypothesised that:

a) EPDS suicidal ideation at 6-8 weeks postpartum is associated with high levels of depressive symptoms at 6-8 weeks

b) EPDS suicidal ideation at 6-8 weeks postpartum is associated with risk factors for depression (young age, unemployment and lack of a partner)

c) EPDS suicidal ideation at 6-8 weeks postpartum is associated with worse outcome 18 weeks later in women being treated for postnatal depression

## 2 Methods

Study design: prospective cohort study nested within an RCT.

### Participants

#### Study population

Postpartum women registered with general practitioners in 77 collaborating practices in the UK located in Bristol (21 practices), London (Croydon and Bromley) (21 practices) and Manchester (35 practices) were eligible for the trial. These practices served a wide range of neighbourhoods including both affluent and socioeconomically deprived urban areas. Recruitment took place between January 2005 and Aug 2007,

Inclusion criteria: recently-delivered women aged 18 years or over who had a live birth and were living with their baby.

Exclusion criteria: women who had a stillbirth or neonatal death; women whose baby was more than 26 weeks old or whose baby had been fostered or adopted; women with psychosis, alcohol or drug abuse; women receiving treatment for depression.

### Recruitment procedure (see figure 1)

**Figure 1:**
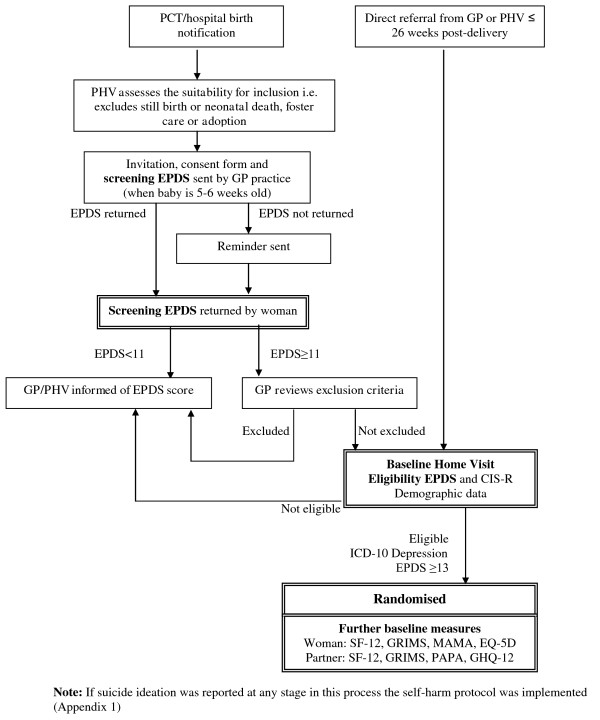
**Summary of RESPOND recruitment procedures**. Flow chart of trial recruitment procedures from initial birth notification to randomization.

#### Initial postal screening

All eligible women were identified by clerical assistants at their registered general practice (primary health care centre) as notification of all births is sent to the practices by maternity services and public health services. Practice health visitors checked that women were eligible before the invitation pack to participate in the main RESPOND trial was sent from the GP just before their baby was six weeks old. This invitation pack included a screening EPDS questionnaire. Responses to the EPDS provided at this stage are used to determine an overall prevalence of suicidal ideation in postnatal women. Only eligible, consenting women who had scored 11 or more on the screening EPDS were invited to participate in a home visit to assess further their eligibility for the main RESPOND trial (see Figure 1).

#### Home visit (baseline)

Once a potentially eligible woman was identified for entry into the main RESPOND trial, a Research Associate would contact the woman and ask if she agreed to a home visit. If a woman consented this was usually conducted at between eight and ten weeks postpartum; however women could receive a home visit up to 26 weeks postpartum. Women completed a baseline EPDS, the revised computerised Clinical Interview Schedule (CIS-R) [17] and a quality of life measure (SF12) (Ware et al, 1996). However, at the start of the trial, women who scored below 13 on the EPDS were not always asked to complete the CIS-R and SF12 so these data are not available for all women who received a home visit. Demographic data were collected by self-report questionnaire.

#### Follow-up schedule

Follow-ups were scheduled at 4 weeks and 18 weeks after the home visit for randomised women participating in the RESPOND trial. At these time points, postal questionnaires were sent to collect outcome measurements (including the EPDS and SF-12).

### Measures

#### Diagnosis of depression and assessment for suicidal ideation (see Additional File 1: appendix 1 for protocol of SI care)

The Edinburgh Postnatal Depression Scale (EPDS) [9], a 10 item self-report questionnaire, was used to screen for postnatal depression. The case definition for probable depression is a score of ≥13. 'Suicidal ideation' (SI) was defined as an answer of 'Sometimes' or 'Yes, quite often' to question 10 of the EPDS 'The thought of harming myself has occurred to me'. 'No suicidal ideation' was defined by answering 'hardly ever' or 'never' for question 10. The EPDS was completed at the screening stage, the baseline home visit, and at the 4 and 18 week follow-ups.

The self-administered computerised version of the CIS-R was used at baseline to obtain a more accurate measure of the woman's clinical state, to confirm a diagnosis of depression. The CIS-R is a fully structured psychiatric assessment for 14 common symptoms of depression and anxiety in the week before interview. Suicidal ideation is asked about in the following questions:

Have you felt hopeless at all during the PAST SEVEN DAYS, for instance about your future? (Yes/No)

In the PAST SEVEN DAYS, have you felt that life isn't worth living? (no/sometimes/always)

In the PAST WEEK, have you thought of killing yourself? (no/yes - but I wouldn't commit suicide/yes)

In the PAST WEEK, Have you thought about a way in which you might kill yourself? (yes/no)

We coded this as 0 (no suicidal thoughts) or 1-4 (endorsement of one, two, three or four of the questions above)

#### Mental and physical health status

Mental and physical health status was assessed at baseline and at both follow-ups and measured using the standard SF-12 version 2 questionnaire [18]. This 12-item measure is a widely used and well validated generic measure of functioning. Mental and physical component scores were calculated using standard algorithms, with higher scores indicating better functioning.

#### Quality of relationship

Quality of relationship was assessed at baseline and at both follow-ups using the Golombok-Rust Inventory of Marital State (GRIMS) [19]. This instrument is a 28-item questionnaire to assess the overall quality of a couple's relationship with higher scores indicating poorer quality of relationship. The measure has been shown to have good reliability (Cronbach's alpha of 0.92 in men and 0.89 in women).

#### Socio-demographic measures

Data were also collected on marital and co-habiting status, educational level, employment status of women and partner, ethnicity and parity.

### Statistical methods

We defined suicidal ideation (SI) in these women as the thought of harming themselves "sometimes" or "quite often" as indicated in the response to question 10 of the EPDS. We defined a 'suicidal case' as measured by the CIS-R as identifying 2 or more items on the CIS-R. The outcome measures investigated in the sub-group of women who participated in the RESPOND trial were the SF-12 physical health subscale, SF-12 mental health subscale and EPDS at 18 weeks.

We estimated the prevalence of SI in the complete sample of all women surveyed at the point of screening, using frequencies and proportions. We investigated the persistence of SI in the sub-group of women who entered the trial and were followed up to 18 weeks, by the association between SI in women at home visit baseline, with SI 4 weeks and 18 weeks later using chi-squared tests for the two outcomes, with and without adjustment for treatment allocation. To assess the agreement between the CIS-R measure of suicidality and EPDS SI in women who had measures of both the CIS-R and the EPDS, we tabulated the two measures on their full scores. We used a chi-square test of the two measures using their full range of categories (0-3 for EPDS and 0-4 for CIS-R) and assessed agreement between suicidal caseness as measured by the CIS-R and EPDS SI using a kappa statistic.

To investigate the ability of SI at baseline to predict outcome at 18 weeks in women who had entered RESPOND study, we first summarised the characteristics (age, ethnicity, marital status, living status, parity, employment, education and health measures) of those with and without SI at baseline, testing for differences using t-tests and chi-squared tests. We employed these measures in a multivariable logistic regression to investigate variables associated with SI in a cross-sectional analysis to be adjusted for as potential confounders in the follow-up analysis. We then investigated baseline predictors of loss to follow up and any factors found to be associated were included in the 18 week analysis. We conducted a linear regression of the clinical outcome measures SF-12 physical health subscale, SF-12 mental health subscale and EPDS at 18 weeks after baseline separately adjusting for the relevant baseline measure, suicidal ideation and treatment allocation and then additionally adjusting for variables associated with baseline suicidal ideation. (No factors were found to be associated with loss to follow-up and so no further adjustment was necessary).

### Ethical approval

The trial was approved by the Scotland Multi-centre Research Ethics Committee (MREC; reference number MREC/03/0/127) and site-specific approval was obtained from the 10 relevant local ethics committees and 10 Primary Care Trusts (PCTs).

## 3 Results

### 3.1 Prevalence and persistence of suicidal ideation

**Figure 2:**
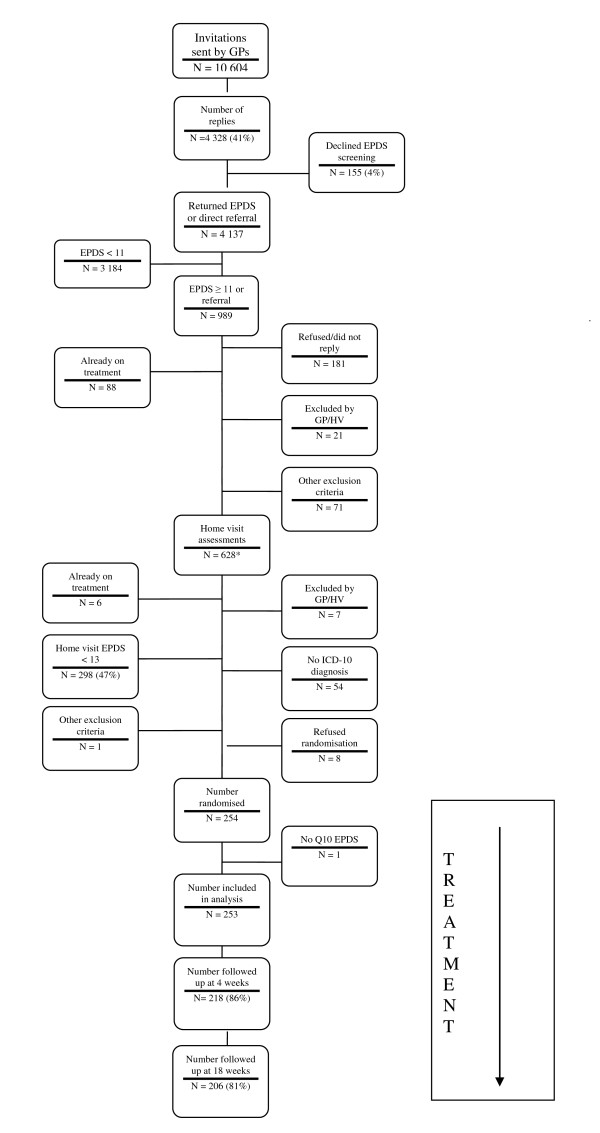
**Flowchart of women through the screening and recruitment procedures in the RESPOND study**. Flow chart of women invited to participate in the RESPOND study, through the screening and recruitment procedures, to follow up at 18 weeks.

4173 women returned the screening questionnaire and of these, a total of 4,150 women completed the EPDS question relating to suicidal ideation (SI) (n = 23, 0.5% missing) (see Figure 2 for flow chart of women through the RESPOND study). Nine percent (95%CI 8.3% -10.1%) (n = 374) reported any suicidal ideation (including hardly ever) and 4% (95% CI 3.2% - 4.4%) reported that the thought of harming themselves had occurred to them sometimes or quite often. The latter group (i.e. reporting that the thought of harming themselves occurred sometimes or often) are defined in this study as experiencing SI. Of those that had SI at home visit and entered the trial (i.e. had a CIS-R primary diagnosis of depression) (n = 32), 56% also reported it at 4 weeks compared to only 9% of those that did not have SI at baseline (p < 0.001 (either unadjusted or adjusted for treatment allocation). At 18 weeks follow-up, only 15% of those with SI at baseline also had it at 18 weeks, which is not significantly different to those without SI at baseline (6%, p = 0.075 adjusted for treatment allocation, (see table 1). No difference in prevalence of SI reporting was seen between the treatment groups at either baseline (p = 0.635), 4 weeks (p = 0.431) or 18 weeks (p = 0.769) follow-up.

**Table 1:** Persistence of Edinburgh Postnatal Depression Scale suicidal ideation (SI) over time

		Suicidal ideation (SI) at home visit		Total	Unadjusted p-value	p-value adjusted for treatment allocation
		No	Yes			
SI at 4 weeks	No	155 (91%)	14 (44%)	169		

	Yes	15 (9%)	18 (56%)	33	< 0.001	< 0.001

	Total	170	32	202		

SI at 18 weeks	No	150 (94%)	29 (85%)	179		

	Yes	9 (6%)	5 (15%)	14	0.065	0.075

	Total	159	34	193		

### 3.2 Validity of EPDS measure of suicidal ideation

We compared the 5 level CIS-R suicidal ideation measure to the 4 level EPDS suicidal ideation measure (table 2) for those women who had a home visit and were administered both measures (n = 331). We also considered dichotomised definitions of "SI cases" as "sometimes/often have suicidal thoughts" on the EPDS and "CIS-R suicidal casesness as endorsement of 2 or more items on the CIS-R. The two measures are significantly associated (chi^2 ^statistic = 145.81, p < 0.001). Almost all (99%) women reporting 'never/hardly ever have suicidal thoughts' on the EPDS question reported positively to 2 or fewer items out of 4 on the CIS-R SI questions. All women who reported significant SI on the EPDS (i.e. often had thoughts of self-harm) reported a score of 2 or more on the CIS-R measure of suicidality. However only 68% of those who "sometimes" considered harming themselves on the EPDS scored positively to 2 or more items on the CIS-R. Defining a suicidal 'case' from the CIS-R as responding positively to 2 or more items agrees with the EPDS definition in 79% of cases and gives a moderate kappa statistic of 0.42.

**Table 2:** Comparison of Clinical Interview Schedule (Revised version) (CIS-R) and Edinburgh Postnatal Depression Scale (EPDS) measures of suicidal ideation at baseline

EPDS Q10	CIS-R					
	
	0	1	2	3	4	Total
**Never**	94	92	23	0	0	209

**Hardly Ever**	9	28	26	3	0	66

**Sometimes**	4	12	21	8	5	50

**Often**	0	0	3	1	2	6

**Total**	107	132	73	12	7	331

### 3.3 Correlates of suicidal ideation

Women who entered the randomised trial (n = 254) (and therefore had an EPDS≥13 and a CIS-R primary diagnosis of depression at home visit) had more detailed sociodemographic and clinical data, enabling us to examine which sociodemographic and clinical variables were associated with SI in this group at baseline. One woman did not complete question 10 of the EPDS and so was not included, leaving 253 women for the remaining analyses (though not all 253 women completed the baseline SF12 - n = 227; ie 90%). There was a small amount of drop-out over the follow-up period, 218 (86%) participants completed the 4 week assessment and 206 (81%) completed the 18 week assessment. Comparisons between those with and without follow-up at 18 weeks indicated no baseline variables were significantly associated with attrition at the 5% level of significance.

In the univariate analysis, we found that women were more likely to experience suicidal ideation at baseline if they were younger, unmarried, unemployed or had a partner that was unemployed. They were also more likely to have a higher score on the GRIMS (reflecting poorer quality of the relationship with partner)and the EPDS and lower (worse) score on the SF-12 mental component (table 3). After multivariate analysis, including all measures (age, ethnicity, marital status, living status, parity, employment, educational level and health measures), adjusted odds ratios remained significant for age and EPDS (see table 3)

**Table 3:** Associations with suicidal ideation (SI) at baseline in women entering the RESPOND trial (ie ICD 10 diagnosis of depression and score of ≥13 on Edinburgh Postnatal Depression Scale) (n = 253)

		All women N = 253	No SI (N = 207)	SI (N = 46)	Unadjusted OR	Adj. OR**	95% CI	p
Age	mean (sd)	28.7 (6.4)	29.1 (6.3)	26.9 (6.5)	0.94 (0.89 - 1.0)	0.78	0.63, 0.97	0.023

Married	Yes	105 (44%)	91 (48%)	14 (31%)				
	No	132 (56%)	100 (52%)	31 (69%)	2.01 (1.01-4.03)	2.39	0.46, 12.45	0.302

Live with partner	Yes	184 (73%)	154 (74%)	29 (64%)				
	No	69 (27%)	53 (26%)	16 (36%)	1.60 (0.81-3.18)	1.06	0.15, 7.22	0.955

Parity	1	96 (38%)	85 (41%)	11 (24%)				
	2	95 (37%)	75 (36%)	19 (41%)	1.96 (0.88-4.38)	4.58	0.74, 28.33	0.129
	3+	63 (25%)	47 (23%)	16 (35%)	2.63 (1.13-6.13)	15.06	1.55, 146. 53	0.019

Highest qualification*	GCSE	67 (27%)	58 (29%)	9 (20%)				
	A-level	32 (13%)	25 (13%)	6 (14%)	1.55 (0.50-4.81)	3.15	0.34, 28.96	0.312
	NVQ	48 (20%)	38 (19%)	10 (23%)	1.70 (0.63-4.56)	1.68	0.24, 11.60	0.601
	Degree	61 (25%)	53 (27%)	8 (18%)	0.97 (0.35-2.70)	10.23	0.88, 119	0.063
	None	36 (15%)	25 (13%)	11 (25%)	2.84 (1.05-7.69)	0.28	0.02, 3.61	0.327

Mother employed	Yes	133 (53%)	118 (58%)	14 (31%)				
	No	117 (47%)	86 (42%)	31 (69%)	3.03 (1.52-6.05)	0.39	0.06, 2.37	0.306

Partner employed	Yes	170 (85%)	146 (89%)	23 (64%)				
	No	31 (15%)	19 (11%)	13 (36%)	4.58 (1.98-10.60)	3.60	0.55, 23.79	0.315

Ethnicity mother	White	196 (78%)	161 (79%)	35 (76%)				
	Non-White	55 (22%)	44 (21%)	11 (24%)	1.15 (0.54-2.45)	1.70	0.21, 13.99	0.623

*Measures at Time 0*								

GRIMS^1^	mean (sd)	15 (6.0)	14 (5.7)	17 (7.0)	1.09 (1.02-1.15)	1.07	0.94, 1.21	0.308

SF12^2 ^- physical	mean (sd)	52.7 (9.4)	53.0 (9.5)	51.9 (8.9)	0.99 (0.95-1.02)	1.04	0.95, 1.14	0.429

SF12 - mental	mean (sd)	28.0 (8.3)	28.7 (8.0)	24.4 (9.3)	0.94 (0.89-0.98)	0.90	0.83, 0.98	0.248

EPDS total^3^	mean (sd)	17.5 (3.4)	16.7 (2.9)	21.1 (3.3)	1.49 (1.32-1.68)	1.96	1.41, 2.73	< 0.001

*GCSEs are secondary school qualifications taken at 16; A-levels are secondary school qualifications taken at 18; NVQs are National Vocational Qualifications for adults or young people

**adjusted for all variables

1. GRIMS - Golombok-Rust Inventory of Marital State

2. SF12 - 12-Item Short-Form Health Survey

3. EPDS - Edinburgh Postnatal Depression Scale

We found no association between suicidal ideation at baseline and any of the three outcomes at 18 weeks - SF-12 physical or mental health or the EPDS total score, when adjusted only for the baseline measure and treatment allocation (Table 4). After additional adjustment for variables associated with suicidal ideation at baseline and loss to follow-up (age, current employment, partner employment and marital status) there was still no association found between suicidal ideation and outcome.

**Table 4:** Suicidal ideation as a predictor of outcome at 18 weeks

Outcome	N	Coefficient of SI	95% CI of coefficient		p-value
**Adjusted for baseline measure of outcome and treatment allocation only**					

**Edinburgh Postnatal Depression Scale total score**	206	-0.57	-2.74	1.61	0.608

**SF-12 Physical Component Score**	160	-2.21	-5.63	1.21	0.204

**SF-12 Mental Component Score**	160	0.96	-3.62	5.54	0.679

**Adjusted for baseline measure of outcome, treatment allocation, age, employment, partner employment, marital status**					

**Edinburgh Postnatal Depression Scale total score**	145	-1.95	-4.74	0.83	0.167

**SF-12 Physical Component Score**	113	-1.79	-5.89	2.31	0.389

**SF-12 Mental Component Score**	113	1.09	-4.52	6.70	0.700

## 4 Discussion

We found that 4% of 4150 women in the community at around 6 weeks postpartum had suicidal ideation (SI) occurring sometimes or quite often, and 9% reported any suicidal ideation. This is a higher prevalence of significant SI than reported by previous studies in Finland and England [5] and this may reflect our study sample which was recruited from areas with higher levels of socioeconomic deprivation than in previous studies [16]. Endorsement of 'yes, quite often' SI on question 10 of the EPDS was associated with affirming at least two CIS-R items on suicidality. However, endorsement of 'sometimes' experiencing SI was not concordant with suicidality as measured by the CIS-R; the kappa statistic of 0.42 reflects this moderate level of agreement. We also confirmed that in women with an EPDS > 12 and a diagnosis of depression, women participating in a treatment trial for postnatal depression were more likely to experience SI if they had more depressive symptoms as measured on the EPDS; in addition they were more likely to be younger, unmarried, unemployed or have an unemployed partner, and have marital problems. In the multivariable analysis, younger age, having 3 or more children and a higher EPDS remained significantly associated with SI. However, SI at baseline was not associated with poorer outcome on follow-up, and this probably reflects the fact that these were women treated for depression in the RESPOND trial (either by medication or psychotherapy).

### Strengths and Limitations

This study is one of the largest studies of suicidal ideation in women in the postpartum period in the literature and the only study to compare the EPDS measure of suicidality with another measure of suicidality (the CIS-R). The main limitation of this study is that, like all previous studies, the EPDS suicidality measure is of self reported thoughts of self harm. Self report may lead to under-reporting of actual suicidal thoughts, though it is also possible that self report may more accurately reflect the truth than clinical interviews. There is some evidence that the rate of SI endorsement on self-administered scales can be considerably higher than on clinician-administered scales in perinatal women [20]. We did not measure suicide attempts (which may be of more clinical importance), and previous research has found that 16.7-27.8% of pregnant women referred to a tertiary clinic for neuropsychiatric evaluation endorsed SI, but only one attempted suicide [20]. Nevertheless, there is evidence that suicide ideators and attempters are a separate but overlapping population from those who die by suicide [21]. There is therefore value in investigating suicidal ideas, particularly as much larger samples would be needed to investigate suicide attempters in an epidemiologically representative population. One final caveat though is that the EPDS question on suicidality (item 10) does not explicitly refer to suicide; rather it asks about harming oneself. As has been pointed out in a review of suicidality in the perinatal period [5], self-harming impulses may or may not reflect intent to die; other dimensions of suicidality, such as reasons for dying and reasons for living, are also needed for more precise risk assessment. The discrepancy found here between women reporting "hardly ever" having thoughts of harming themselves on the EPDS but on the CIS-R reporting that they had thought of killing themselves in the last week, suggests that women are not equating the two statements, and item 10 of the EPDS therefore may not be the best measure of suicidality.

Other limitations include possible selection bias - our estimate of prevalence of SI in the screened population was carried out in women not receiving treatment for depression and this selection bias needs to be borne in mind when interpreting our results. In addition, as with most other studies in this area, the analyses of correlates of SI were limited by the small numbers reporting SI. Finally there are limits on the external validity of this study as our findings on persistence and correlates of SI were carried out on the women who entered the RESPOND trial and may not be generalisable to women outside of a treatment trial.

### Implications

The clinical significance of SI as measured by the EPDS is not entirely clear, as discussed above. Nevertheless, endorsement of the 'often' experiencing SI item on EPDS was associated with at least two of the CIS-R items of suicidality. Although it remains unclear whether the EPDS should be used routinely in perinatal practice [14], maternity and community services internationally use this instrument to screen for perinatal depression. The endorsement of question 10 on suicidal ideation can cause major concern for healthcare professionals in these settings who do not usually have mental health training and do not know how to address this in their clinical practice. They may also be concerned about the impact on patients of being asked such a question and fear that asking a question about suicidal thoughts could 'induce' suicidal thoughts and behaviour; however a recent RCT of screening for suicidal ideation in primary care found no evidence to support the view that such screening leads to an increase in feelings that life is not worth living [22].

This study suggests that these professionals should be aware that endorsement of "often" will usually mean there is significant suicidality and depressive symptomatology warranting referral to an appropriate professional (e.g. general practitioner) for further assessment. However, suicidal ideation does not appear to predict poor outcomes in women who are treated for depression. Women with depressive symptoms and suicidal ideation should benefit from appropriate treatment for postnatal depression such as health visitor delivered non-directive counselling, cognitive behavioural therapy or antidepressants [16]. Women may have strong preferences regarding these treatments and where possible their preferred treatment should be offered as this may improve outcome further [16,23,24].

## Conclusion

Healthcare professionals using the EPDS should be aware of the significant suicidality that is likely to be present in women endorsing 'yes, quite often' to question 10 of the EPDS. However, suicidal ideation does not appear to predict poor outcomes in women being treated for postnatal depression.

## 5 Competing interests

The authors declare that they have no competing interests.

## 6 Authors' contributions

DS conceived the study design and was the chief investigator for the RESPOND trial. AM collected the data at the London centre and helped to draft the manuscript. AT participated in the design and coordination of the study. CF performed the statistical analysis and helped draft the manuscript. LMH participated in the design and coordination of the study and led the writing of the manuscript. All authors approved the final manuscript.

## Pre-publication history

The pre-publication history for this paper can be accessed here:

http://www.biomedcentral.com/1471-2393/11/57/prepub

## Supplementary Material

Additional file 1 **Appendix 1**. Protocol for responding to women reporting SI. Click here for file

## References

[B1] Lewis G (ed) The Confidential Enquiry into Maternal and Child Health (CEMACH). Saving Mothers' Lives: reviewing maternal deaths to make motherhood safer - 2003-2005. The Seventh Report on Confidential Enquiries into Maternal Deaths in the United Kingdom 2007 London: CEMACH

[B2] Austin MP Kildea S Sullivan E Maternal mortality and psychiatric morbidity in the perinatal period: challenges and opportunities for prevention in the Australian setting Med J Aust 2007 186 364 367 17407434 10.5694/j.1326-5377.2007.tb00940.x

[B3] Moller HJ Suicide, suicidality and suicide prevention in affective disorders Acta Psychiatr Scand 2003 418 73 80 12956819

[B4] Bernal M Haro JM Bernert S Brugha T de Graaf R Bruffaerts R Lépine JP de Girolamo GE Vilagut G Gasquet I Torres JV Kovess V Heider D Neeleman J Kessler R Alonso J Risk factors for suicidality in Europe: Results from the ESEMED study J Affect Disorders 2007 101 27 34 10.1016/j.jad.2006.09.018 17074395

[B5] Lindahl V Pearson J Colpe L Prevalence of suicidality during pregnancy and the postpartum Arch Womens Ment Health 2005 8 2 77 87 10.1007/s00737-005-0080-1 15883651

[B6] Welch SS A review of the literature on the epidemiology of parasuicide in the general population Psychiatr Serv 2001 52 368 375 10.1176/appi.ps.52.3.368 11239107

[B7] King RA Schwab-Stone M Flisher AJ Greenwald S Kramer RA Goodman SH Lahey BB Shaffer D Gould MS Psychosocial and risk behavior correlates of youth suicide attempts and suicidal ideation J Am Acad Child Adolesc Psychiatry 2001 40 837 846 10.1097/00004583-200107000-00019 11437023

[B8] Lish JD Zimmerman M Farber NJ Lush DT Kuzma MA Plescia G Suicide screening in a primary care setting at a veterans affairs medical center Psychosom 1996 37 413 424 10.1016/S0033-3182(96)71528-1 8824120

[B9] Cox JL Holden JM Sagovsky R Detection of postnatal depression. Development of the 10-item Edinburgh Postnatal Depression Scale Br J Psychiatry 1987 150 782 786 10.1192/bjp.150.6.782 3651732

[B10] Beck AT Steer RA Manual for the Beck Depression Inventory 1987 San Antonio, TX: Psychological Corporation

[B11] Pinheiro RT da Silva RA Magalha˜ es PVS Horta BL Pinheiro KAT Two studies on suicidality in the postpartum Acta Psychiatr Scand 2008 118 160 163 10.1111/j.1600-0447.2008.01184.x 18498435

[B12] Gibson J McKenzie-McHarg K Shakespeare J Price J Gray R A systematic review of studies validating the Edinburgh Postnatal Depression Scale in antepartum and postpartum women Acta Psychiatr Scand 2009 119 5 350 364 10.1111/j.1600-0447.2009.01363.x 19298573

[B13] Matthey S Using the Edinburgh Postnatal Depression Scale to screen for anxiety disorders Depress Anxiety 2008 25 11 926 931 10.1002/da.20415 18041072

[B14] NICE: Antenatal and postnatal mental health The NICE guideline on clinical management and service guidance 2007 The British Psychological Society. Gaskell 21678630

[B15] Bick D Howard LM When should women be screened for postnatal depression? Expert Review Neurotherapeutics 2010 10 2 151 154 10.1586/ern.09.156 20136374

[B16] Sharp DJ Chew-Graham CA Tylee A Lewis G Mulligan J Howard LM Anderson I Abel K Turner KM Tallon D McCarthy A Peters TJ A pragmatic randomised controlled trial to compare antidepressants with a community based psychosocial intervention for the treatment of women with moderate postnatal depression: The RESPOND trial Health Technol Assess 2010 14 43 10.3310/hta14430 20860888

[B17] Lewis G Pelosi AJ Araya R Dunn G Measuring psychiatric disorder in the community: a standardized assessment for use by lay interviewers Psychol Medicine 1992 22 465 486 10.1017/S0033291700030415 1615114

[B18] Ware JE Kosinski M Turner-Bowker DM Sundaram M Gandek B Mariush ME SF-12v2^® ^Health Survey: administrative guide for clinical trial investigators 2009 Lincoln, RI: QualityMetric Incorporated [See also: Ware JE, Kosinski M, Keller SD. A 12-Item Short-Form Health Survey: construction of scales and preliminary tests of reliability and validity. Med Care. 1996, 34: 220-33

[B19] Rust J Bennun I Crowe M Golombok S The GRIMS. A psychometric instrument for the assessment of marital discord J Family Ther 1990 12 45 57 10.1046/j..1990.00369.x

[B20] Newport DJ Levey LC Pennell PB Ragan K Stowe ZN Suicidal ideation in pregnancy: assessment and clinical implications Arch Womens Ment Health 2007 10 5 181 7 10.1007/s00737-007-0192-x 17726640

[B21] Beautrais AL Suicides and serious suicide attempts: Two populations or one? Psychol Med 2001 31 837 895 11459381 10.1017/s0033291701003889

[B22] Crawford M Thana L Methuen C Ghosh P Stanley SV Ross J Gordon F Blair G Bajaj P Impact of screening for risk of suicide: randomised controlled trial Br J Psychiatry 2011 198 379 384 10.1192/bjp.bp.110.083592 21525521

[B23] Howard LM Thornicroft G Patient Preference Randomised Controlled Trials in Mental Health Research Br J Psychiatry 2006 188 303 304 10.1192/bjp.188.4.303 16582054

[B24] Howard LM Flach C Leese M Byford S Killaspy H Cole L Lawlor C Betts J Cutting P McNicholas S Sharac J Johnson S The effectiveness and cost effectiveness of admissions to women's crisis houses compared with traditional psychiatric wards - a pilot patient preference randomized controlled trial Br J Psychiatry 2010 197 s32 s40 10.1192/bjp.bp.110.081083 20679277

