# Supplementary material for: The prevalence of suicidal ideation identified by the Edinburgh Postnatal Depression Scale in postpartum women in primary care: findings from the RESPOND trial
Source: BMC Pregnancy Childbirth. 2011 Aug 3;11:57. doi: 10.1186/1471-2393-11-57 (PMC3161003; doi:10.1186/1471-2393-11-57)
Supplement: Additional file 1 — Appendix 1. Protocol for responding to women reporting SI. [file 1471-2393-11-57-S1.DOC]

**APPENDIX 1 Protocol for responding to women reporting SI**

RESPOND SELF-HARM ACTION PRO FORMA

Risk of self-harm is defined as a score of 2 or 3 in the relation to question 10 on the EPDS or the outcome of the suicide intent questions on the CIS-R is one of the following:

1. CIS-R SUICIDE INTENT: Patient feels life isn’t worth living
2. CIS-R SUICIDE INTENT: Patient has had suicidal thoughts
3. CIS-R SUICIDE INTENT: Patient has had suicidal plans

If at any point during the study period risk of self-harm has been expressed by a woman either in postal, telephone or face-to-face interview then the following action must be taken and recorded by the researcher:

Postal questionnaire

Contact patient by telephone to discuss

Woman contacted?

NO

Discuss with P.I.

YES

Face-to-face or telephone interview

Advise woman to discuss these thoughts with HV/GP

Has GP been informed previously?

NO

Ask for permission to inform GP

Does woman agree?

NO

“As you’ve had these thoughts, I need to let my clinical colleague know who may telephone you”

Inform PI or nominated deputy who may contact patient to assess risk or decide to break confidentiality and contact GP.

YES

Research to make notify woman’s GP to discuss given option to withdraw woman.

YES

Notify PI or nominated deputy of action taken.
